# Maternal and Neonatal Outcomes of COVID-19 Infection in Pregnancy

**DOI:** 10.34172/aim.2023.07

**Published:** 2023-01-01

**Authors:** Maryam Vizheh, Maryam Allahdadian, Hatav Ghasemi-Tehrani, Salut Muhidin, Maryam Hashemi, Maryam Dehghan

**Affiliations:** ^1^Australian Institute of Health Innovation (AIHI), Faculty of Medicine, Health and Human Sciences, Macquarie University, Sydney, New South Wales, 2109, Australia; ^2^Department of Reproductive Health and Midwifery, School of Nursing and Midwifery, Tehran University of Medical Sciences, Tehran, Iran; ^3^Department of Midwifery, Nursing & Midwifery Faculty, Falavarjan Branch, Islamic Azad University, Isfahan, Iran; ^4^Fertility Department, School of Medicine, Isfahan University of Medical Science, Isfahan, Iran; ^5^Department of Management, Macquarie Business School, Macquarie University, New South Wales, 2109, Australia; ^6^Department of Obstetrics and Gynecology, School of Medicine, Isfahan University of Medical Sciences, Isfahan, Iran

**Keywords:** COVID-19, Infectious, Severe acute respiratory syndrome coronavirus 2 (S, Maternal mortality, Neonates, Pregnancy outcome

## Abstract

**Background:**

Limited data is available on the full spectrum of maternal COVID-19 infection in terms of pregnancy outcomes. The present study aimed to compare the maternal and neonatal outcomes of COVID-19 in infected and non-infected pregnant women.

**Methods:**

A dual-site retrospective cohort study was conducted in two tertiary hospitals in Isfahan, Iran. The sample included 104 infected and 210 non-infected hospitalized pregnant women. Odds ratios (OR) were estimated using multivariate logistic regression.

**Results:**

There were significant differences between COVID-19-infected and non-infected pregnant women regarding preterm labor (PTL) (odds ratio [OR]: 11.34, 95% confidence interval [CI]: 1.19–48.54, *P*=0.035); hospitalization days (OR: 7.21, 95% CI: 4.05–12.85, *P*≤0.001); cesarean section (CS) (OR: 4.76, 95% CI: 1.78–12.45, *P*=0.002); neonatal admission to neonatal intensive care unit (NICU) (OR: 1.28, 95% CI: 1.12–1.67, *P*=0.004); and neonatal respiratory distress (OR: 2.37, 95% CI: 1.02– 5.47, *P*=0.044). No significant association was found between COVID-19 infection and abortion (OR: 0.06, 95% CI: 0.01–1.45, *P*=0.084); stillbirth (OR: 1.84, 95% CI: 0.05–39.68, *P*=0.743); Apgar score (1 minute) (OR: 0.91, 95% CI: 0.74–1.13, *P*=0.382); Apgar score (5 minutes) (OR: 0.97, 95% CI: 0.81–1.18, *P*=0.765); and low birth weight (LBW) (OR: 4.76, 95% CI: 1.78–12.45, *P*=0.002).

**Conclusion:**

PTL, CS, neonatal admission in NICU, neonatal respiratory distress, and hospitalization days were significantly higher in pregnant women with COVID-19 compared to those without infection.

## Introduction

 The novel coronavirus pneumonia (COVID-19), a highly infectious disease, has been declared a public health emergency of international concern.^1^ Although many efforts have been made to restrict the epidemic, the infection is still spreading fast all over the world. This pandemic has led to dramatic consequences, including loss of human life worldwide and unprecedented challenges to public health such as maternal and child health.^2^ Currently, limited data is available on the full spectrum and impact of maternal COVID-19 infection on pregnancy outcomes.^3^ Partial immune suppression, as well as the physiological changes in the cardiovascular and respiratory systems, may endanger pregnant women compared to the general population in viral infections such as COVID-19.^4^ Previous viral epidemics such as severe acute respiratory syndrome (SARS), the Middle East respiratory syndrome (MERS), Ebola, H1N1, and influenza-A showed higher mortality and morbidity in pregnant women compared to the non-pregnant.^5^ Evidence shows that viral pneumonia in pregnancy is associated with poor obstetrical outcomes, including preterm labor (PTL), low birth weight (LBW), fetal growth retardation, low Apgar score < 7 at 5 minute, and perinatal mortality compared to pregnant women without pneumonia.^6^ Therefore, it has been hypothesized that COVID- 19 infection may deteriorate pregnancy outcomes and maternal and neonatal health and surveillance.^7^

 Challenges resulting in pregnancies due to COVID-19 have led to continuous updating of management protocols.^8^ Liang andAcharya emphasize the importance of reporting all cases of COVID-19 pregnancies to achieve transparent and comprehensive data on the effect of COVID-19 in pregnancy.^7^ Continually acquiring all detailed information of COVID -19 in pregnant cases helps draw clear conclusions by which accessing concrete results to generate evidence and guidance of clinical management would be more feasible.^8^

 We need to provide further information to conclude whether or not infected pregnant women have similar maternal and neonatal outcomes compared to the non-infected pregnant. Hence, this study aimed to report the demographic, epidemiological, clinical, and paraclinical characteristics and compare the maternal and neonatal consequences of pregnant women with and without COVID-19 infection.

## Materials and Methods

###  Study Design and Participants

 This is a retrospective dual-site cohort study including hospitalized women in two hospitals: Amin and Alzahra, affiliated with Isfahan University of Medical Sciences, Isfahan, Iran. The observations were conducted between March 2020 and September 2020.

 We enrolled pregnant women whose COVID-19 infection was confirmed either by real-time polymerase chain reaction (RT-PCR) or computed tomography (CT) Scan. To compare the maternal and neonatal outcomes, pregnant women with negative RT-PCR tests or without typical clinical symptoms were chosen as the control group. In Iran, universal screening is not usually performed at the time of admission, so the exclusion criteria were precisely assigned to avoid entering asymptomatic people in the control group. For this purpose, in the control group, if women had negative CT scan or SARS-CoV-2 RT-PCR test but had typical clinical symptoms or para-clinical results such as lymphopenia (lymphocyte count <1.0 × 109/L), or increased C-reactive protein (CRP) ( ≥ 10 mg/L), they were excluded from the study.

###  Data Collection

 All patients consecutively admitted to two hospitals in Isfahan, Iran, between March 2020 and September 2020 were included. The authors applied a customized data collection form for every patient to avoid missing data. To ensure data accuracy, retrieved data were reviewed by two authors (MV and MA) independently.

###  Outcomes and Definitions

 In this study, we evaluated all demographic and medical risk factors; clinical symptoms and paraclinical results; maternal outcomes including maternal death, admission to the intensive care unit (ICU); cardiopulmonary resuscitation (CPR); treatment; management; obstetrical consequences including PTL, LBW, cesarean section (CS), fetal distress; postpartum complications such as hemorrhage, fever; neonatal outcomes including death, infection, respiratory distress, Apgar score at 1 and 5 minutes, and poor feeding.

###  Statistical Analysis

 SPSS, version 24, was used for analyzing the data. Categorical variables are expressed as numbers (%), and continuous variables are expressed as ranges. To compare infected and non-infected pregnant women, Student’s* t*-test was used for continuous variables, and chi-square and Fisher’s exact test were applied for categorical variables. We estimated the odds ratios (ORs) and 95% confidence intervals (CIs) using the multivariate logistic regression to adjust the effect of some potential confounders which were significantly different between the two groups at the baseline, including gestational age, medical history (anemia, hypothyroidism, gestational diabetes mellitus), and drug history (using ASA, enoxaparin, levothyroxine, insulin), between the two groups.

## Results

###  Study Population

 During the study period, 617 pregnant women were admitted for either delivery or obstetric complications to the two mentioned hospitals in Isfahan province. Among them, 104 were positive for SARS-CoV-2 infection through either the RT-PCR SARS-CoV-2 test or CT scan and were considered the infected group. As mentioned in the method section, due to lack of universal COVID-19 screening among pregnant women in Iranian hospitals, meticulous criteria were considered to minimize the possibility of entering infected women into the control group. For this reason, all pregnant women whose RT-PCR and CT-scan were negative or had not been performed but showed at least one possible symptom of COVID-19 infection such as fever, cough, myalgia, headache, rhinorrhea, chill, etc., or had suspected laboratory findings in favor of a possible COVID-19 infection, including lymphopenia or increased CRP were excluded from the study. Consequently, 280 women were excluded. On the other hand, 13 women were excluded because of missing essential data in their medical records. Eventually, 210 non-infected women were included in the control group.

 As illustrated in Table 1, there were no significant differences between the two groups of infected and non-infected pregnant women regarding maternal age, gravida, parity, abnormal BMI (BMI > 30 kg/m^2^), diabetes, hypertension, and preeclampsia. However, infected pregnant women showed a higher percentage of hypothyroidism and gestational diabetes mellitus. Using medicines including ASA, Enoxaparin, Levothyroxine, and Insulin was significantly higher in COVID-19 group. Maternal age in both infected and non-infected groups was 27.77 ± 5.02 and 28.96 ± 4.33, respectively (*P* = 0.07). While both groups had similar gravidity (1.7 ± 0.66 vs. 1.88 ± 0.84, *P*= 0.06), gestational age in infected women was significantly lower than the control group (26.32 ± 9.21 vs. 31.40 ± 10.45, *P*< 0.001).

**Table 1:** Comparison of Maternal Characteristics and Comorbidities of Infected and Non-infected Pregnant Women

**Characteristics**	**Infected Pregnant Women** **(n=104)**	**Non-infected Pregnant Women (n=210)**	***P *** **Value**
Baseline			
Age (y)	27.77 ± 5.02	28.96 ± 4.33	0.07
Gestational Age (wk)	26.32 ± 9.21	31.40 ± 10.45	<0.001
Gravidity	1.7 ± .66	1.88 ± 0.84	0.06
Parity	0.47 ± .71	0.72 ± .68	0.059
Risk factors and comorbidities			
Prevalence of presence of comorbidities	42 (40.4%)	61 (29.04%)	0.02
BMI	24.51 ± 5.16	24.56 ± 3.41	0.86
Abnormal BMI (> 30 kg/m^2^)	14 (13.5%)	16 (7.6%)	0.097
Gestational Diabetes Mellitus	8 (7.7%)	4 (1.9%)	0.012
Diabetes mellitus	1 (1.0%)	1 (0.5%)	0.611
Hypothyroidism	24 (23%)	27 (15.7%)	0.021
Hypertension	3 (2.9%)	4 (1.9%)	0.580
Preeclampsia	4 (3.8%)	4 (1.9%)	0.304
Anemia	9 (8.65%)	2 (1.0%)	<0.001
Drug history			
ASA	22 (21.1%)	15 (7.1%)	<0.001
Enoxaparin	18 (17.3%)	1 (0.5%)	<0.001
Levothyroxine	26 (25.0%)	28 (13.3%)	0.001
Insulin	7 (6.7%)	2 (0.9%)	0.004
Metformin	4 (3.8%)	6 (2.8%)	0.04

###  Clinical Presentations 

 Clinical presentations in the infected group are presented in Table 2. Weakness (88/104, 84.6%) and lethargy (87/104, 83.7%) were the most common symptoms, followed by dyspnea (61/104, 58.7%) and chest pain (54/104, 51.9%). Fever, myalgia, cough, and anosmia were the other common symptoms reported by pregnant patients (Table 2).

**Table 2:** Clinical Symptoms of 104 COVID-19 Infected Pregnant Women

**Common Symptoms at Onset **	**Results, No. (%)**
Weakness	88 (84.6)
Lethargy	87 (83.7)
Anosmia	46 (44.2)
Fever	50 (48.1)
Cough	49 (47.1)
Dyspnea	61 (58.7)
Myalgia	49 (47.1)
Sore throat	24 (23.1)
Shortness	41 (39.4)
Headache	36 (34.6)
Chill	21 (20.2)
Diarrhea	19 (18.3)
Nausea	21 (20.2)
Vomit	17 (16.3)
Loss of appetite	28 (26.9)
Ageusia	40 (38.5)
Running nose	37 (35.6)
Agitation	30 (28.8)
thrombosis	12 (11.5)
Chest pain	54 (51.9)

 The most common blood groups in women with and without COVID-19 infection were A positive (42, 46%) and B positive (62, 29.5%) (*P*= 0.03). The most frequent abnormalities in laboratory tests in infected women were a rise in creatinine (0.73 ± 0.12 vs. 0.60 ± 0.07, *P* < 0.001), ESR (37.76 ± 15.62 vs. 7.99 ± 2.58, *P* < 0.001), CRP (52.41 ± 27.03 vs. 4.65 ± 3.48, *P* < 0.001), neutrophils (71.37 ± 7.21 vs. 60.94 ± 4.93, *P* < 0.001) as well as lymphopenia (16.68 ± 5.03 vs. 23.60 ± 3.15, *P* < 0.001). Infected pregnant women demonstrated higher heart rate (111.88 ± 13.71 vs. 78.52 ± 8.15, *P* < 0.001) and respiratory rate (0.73 ± 0.12 vs. 0.60 ± 0.07, *P* < 0.001) and lower O_2_ saturation (89.86 ± 9.95 vs. 96.75 ± 2.80, *P* = 0.00) than non-infected pregnant women (Table 3).

**Table 3:** Paraclinical Analysis of Study Population

**Characteristics **	**Infected Pregnant Women** **(n=104)**	**Non-infected Pregnant Women** **(n=210)**	***P *** **Value**
Hemoglobin (g/L)	11.72 ± 1.32 (6.80–16.20)	12.02 ± 1.09 (8.50-13.70)	0.033
Neutrophils (%)	71.37 ± 7.21 (60.00–87.40)	60.94 ± 4.93 (44.00–72.20)	<0.001
Lymphocytes (%)	16.68 ± 5.03 (6.50–26.00)	23.60 ± 3.15 (18.00–32.00)	<0.001
Urea (mg/dL)	9.39 ± 3.00 (6.50–20.00)	9.41 ± 1.26 (6.00–14.00)	0.951
Creatinine (mg/dL)	0.73 ± 0.12 (0.6-.1.3)	0.68 ± 0.07 (0.60-1.1)	<0.001
ESR (mm/h)	37.76 ± 15.62 (18.00–70.00)	7.99 ± 2.58 (4.00–16.00)	<0.001
CRP (mg/L)	52.41 ± 27.03 (17.00–135.00)	4.65 ± 3.48 (3.00–21.00)	<0.001
Platelets ( × 10^3^/mL)	177.82 ± 56.96 (71.00–336.00)	188.30 ± 50.11 (87.00–336.00)	.097
Systolic pressure	106.89 ± 11.87 (80.00–150.00)	109.29 ± 13.47 (90.00–190.00)	0.067
Diastolic pressure	65.34 ± 10.84 (40.00–90.00)	67.52 ± 8.93 55.00–110.00	0.087
Heart number	111.88 ± 13.71 (86.00–194.00)	78.52 ± 8.15 (66.00–98.00)	<0.001
Respiratory rate	23.13 ± 4.84 (16.00–40.00)	18.09 ± 1.10 (16.00–21.00)	<0.001
O_2_saturation	89.86 ± 9.95 (40.00–97.00)	96.75 ± 2.80 (94.00–98.00)	<0.001

ESR, Erythrocyte sedimentation rate; CRP, C-reactive protein; CT, computed tomography; CXR, chest X-ray.

 A combination of various treatments including high-flow nasal cannula oxygen, hydroxychloroquine, chloroquine, antiviral treatments (oseltamivir, ribavirin, etc.), antibiotics, and corticosteroids was administrated to pregnant women with infection.

###  Maternal and Neonatal Outcomes

 Of the 104 infected pregnant, there were three cases of abortion (2.9%), two intra-uterine fetal deaths (1.9%), and four stillbirths (3.8%), 60 delivered their babies (57.7%), and 41 pregnancies (39.4%) continued. In the control group, 134 (63.8%) women delivered their babies, 15 (7.1%) had an abortion, and 61 (29%) women continued their pregnancies. Forty-one pregnancies (39.4%) in the infection group and 61 (29%) in the control group continued their pregnancies (Table 4). We did not follow these cases because most women with COVID-19 or women with obstetric complications in the control group were referred from various cities in the Isfahan province to these tertiary hospitals to receive special medical care, and then they returned to their hometowns to continue their pregnancies. So, they were not necessarily from the Isfahan city where this research was conducted. Access to these patients was difficult for the researchers due to time and resource constraints. Consequently, we did not follow up on these cases.

**Table 4:** Summary of Logistic Regression Analysis Results of Maternal and Neonatal Outcomes in Two Groups of Infected and Non-infected Pregnant Women**

**Pregnancy Characteristics**	**Infected Pregnant Women** **(n=104)**	**Non-infected Pregnant Women** **(n=210)**	OR* **(95% CI)**	***P *** **Value**
Delivery	60 (57.7%)	134 (63.8%)	0.28 (0.02–4.77)	0.379
Continued pregnancy	41 (39.42%)	61 (29.0%)	0.56 (0.03–9.26)	0.682
Abortion	3 (2.9%)	15 (7.14%)	0.06 (0.01–1.45)	0.084
Stillbirth	4 (3.8%)	1 (0.5%)	1.84 (0.05–39.68)	0.743
Preterm Labor	11 (10.58%)	16 (7.6%)	11.34 (1.19–48.54)	0.035
Mode of delivery (N)				
NVD	16 (15.4%)	93 (44.3%)	—	(Ref)
CS	44 (42. 3%)	40 (19.1%)	4.76 (1.78–12.45)	0.002
Hospitalization days	6.47 ± 1.5	2.05 ± 1.26	7.21 (4.05–12.85)	<0.001
Neonatal outcomes				
Apgar score (1 min)	6.21 ± 3.31	8.25 ± 1.69	0.91 (0.74–1.13)	0.382
Apgar score (5 min)	7.63 ± 3.89	9.56 ± 1.76	0.97 (0.81–1.18)	0.765
Low birth weight (**< **2500 g)	20 (19.2%)	18 (8.6%)	1.05 (0.39–2.83)	0.921
Neonatal admission to NICU	8 (7.7%)	4(1.9%)	1.28 (0.12–0.67)	0.004
Neonatal respiratory distress	7 (6.7%)	3 (1.4%)	2.37 (1.02–5.47)	0.044

NVD, normal vaginal delivery; CS, cesarean section. *OR, Infected/Non-Infected; **Multivariate logistic regression with adjusted variables: hypothyroidism, anemia, medicines (ASA, enoxaparin, levothyroxine, insulin, metformin).

 Given the significant differences between the two groups at the baseline in gestational age, maternal comorbidities, and drug history, multivariate logistic regression was used to adjust these potential confounders. Compared with non-infected pregnant women, infected pregnant women were more likely to have a preterm delivery (OR: 11.34, 95% CI: 1.19–48.54, *P* = 0.035). The CS rate was significantly higher in infected patients (OR: 4.76, 95% CI: 1.78–12.45, *P* = 0.002). The main indication for CS in infected women was COVID-19 (26, 25.0%) compared with the previous history of CS (24, 11.4%) in the control group. Moreover, women giving birth with COVID-19, compared to those without COVID-19, were more likely to have a prolonged stay at the hospital (OR: 7.21, 95% CI: 4.05–12.85, *P* ≤ 0.001). Neonatal admission to the neonatal intensive care unit (NICU) (OR: 1.28, 95% CI: 1.12–1.67, *P* = 0.004); and neonatal respiratory distress (OR: 2.37, 95% CI: 1.02–5.47, *P* = 0.044) were significantly higher in pregnant women with COVID-19 compared to the control group (Table 4).

 After adjusting for potential confounders, no significant association was found between COVID-19 infection and LBW (OR: 1.05, 95% CI: 0.39–2.83, *P* = 0.921). There was also no significant difference in both groups in terms of Apgar score (1 minute)(OR: 0.91, 95% CI: 0.74–1.13, *P* = 0.382); Apgar score (5 minute) (OR: 0.97, 95% CI: 0.81–1.18, *P* = 0.765) (Table 4).

 On the other hand, some postpartum complications were observed in infected women. Four women experienced thrombosis (3.8%), while no cases were seen in the control group. Postpartum fever and hemorrhage were detected in 9 (8.7%) and 10 (9.6%), which were higher than the control group (1, 0.5%, and 5, 2.4%, respectively). Moreover, respiratory distress was detected in 13 (12.5%) infected women after delivery. Of the 104 pregnant infected women, 15 were admitted to the ICU (10.6%), three needed a mechanical ventilator (2.9%), three developed organ failure (2.9%), nine needed CPR (8.6%), and ten died (9.6%). None of the mothers in the control group needed ICU admission or CPR or died. However, the number of these cases was so low that performing adjustment based on the cofounder factors was impossible. So, these data should be interpreted cautiously.

## Discussion

 This study was conducted to assess whether COVID-19 puts pregnant women at risk of developing adverse maternal and neonatal outcomes.

 In Iran, the first official announcement of COVID-19 infection was on February 19, 2020. According to Iran’s ministry of health, in October 2021, more than five million confirmed cases and 121 000 deaths have occurred during this pandemic in all provinces of Iran.^9^ This study was conducted in the Isfahan province, located in the central area of the country with more than five million inhabitants. The city of Isfahan, its provincial capital, is the third-most populous city in Iran. Over the pandemic period, especially during the early pandemic, Isfahan experienced a dramatic rise in the number of infected people. One of the reasons for this increase was the proximity of Isfahan to the Qom province, which was the first place to identify the virus in the country.

 This study’s results showed that maternal and neonatal morbidity and mortality in pregnant women with COVID-19 infection are considerable. The clinical manifestations of COVID-19 are partly different from other studies. Several studies have reported that fever and cough were the main symptoms of COVID-19 in pregnant patients.^10^ In one study, a persistent cough (80%) and chest pain (73%) were the most common symptoms in hospitalized pregnant women compared to anosmia (63%) and headache (72%), the most frequent symptoms among non-hospitalized ones.^11^ However, in the present study,weakness (84.6%) and lethargy (83.7%) were the most common symptoms among infected pregnant women. There might be several reasons for this.The number of COVID-19 infected people in Iran was so high that screening all suspected people was impossible. Moreover, some patients with mild symptoms were simply asked to rest at home and were referred to the hospital in the case of aggravating symptoms. However, pregnant women are a priority in the health system and often receive regular care in health centers. They are more likely to be screened or hospitalized with even mild symptoms. This situation increases the possibility of early diagnosis of the disease. Therefore, it is less likely that they show classic symptoms of infection.^12^

 Primary investigations published in the early pandemic, which were mostly on women in the third trimester of pregnancy and with a small sample size, suggested that COVID-19 does not impose a higher risk of complications on pregnant women. However, the new studies reported that women in pregnancy and postpartum are at a higher risk of adverse consequences of infection.^13^ Di Mascio et al, in a systematic review assessing pregnant women infected by SARS, MERS, and COVID-19, concluded that COVID-19 is associated with even worse consequences, including a higher rate of PTL, preeclampsia, cesarean, and perinatal death.^14^ Similarly, this study found out that more than one-tenth of pregnancies in the infected group were preterm. Consistent with these observations, a study in France concluded that women with symptomatic COVID-19 showed significantly higher rates of PTL (7.2% vs.16.9%, *P* = 0.003).^15^ In this study, infected mothers were significantly more likely to have a CS than the control group. Although there is no evidence supporting performing CS to prevent the vertical transition of infection from mother to fetus, the rate of CS is relatively high in many studies,^16,17^ including in this current research. This is primarily because of obstetric complications frequently reported in the pregnancies affected by COVID-19, such as fetal distress, premature rupture of membranes (PROM), etc.^18^ However, as much as possible, it should be restricted to obstetric indications.^19^

 In a systematic review including 9032 pregnant women with COVID-19 and 338 infants, 2% of neonates were positive for SARS-CoV-2, and 2% died.^20^ In the present study, three neonates (2.9%) died, one of whom tested positive for the SARS-CoV-2, and the others were not tested as they died immediately after birth. In Iran, all neonates born to mothers with COVID-19 get tested immediately after birth to detect SARS-CoV-2. All neonates born to infected mothers were immediately isolated and separated from their mothers and also from midwives and doctors. Restricted precautions were considered to minimize transmission of infection. However, it cannot affirm the possibility of vertical transmission because no tests were done on pregnancy products such as amniotic fluid, umbilical cord, or breast milk. A study in India showed that 10.7% of neonates born to infected mothers were positive for SARS-CoV-2, although they did not show severe consequences.^21^

 In line with the results of the present study, an overview of systematic reviews showed similar Apgar scores and stillbirth in infected and non-infected groups.^22^ Among adverse neonatal outcomes, higher rates of neonatal respiratory distress syndrome and admission to the NICU were reported frequently.^22,23^ A possible reason for this higher rate of NICU admission can be due to more precise investigation and monitoring of neonates born to infected mothers^24^ and also the higher rates of PTL.

 In the present study, four women, all in the infected group, developed postpartum thrombosis, and all died. Pregnancy itself predisposes the risk of prothrombotic conditions, so pregnant women may be at a higher risk of coagulopathic and thromboembolic complications associated with COVID-19 infection.^25^ Similarly, a systematic review showed a higher rate of coagulopathy and thromboembolism in pregnancy with COVID-19 infection.^26^ Detecting these conditions in pregnant patients and close observation may be effective in minimizing maternal morbidity and mortality due to COVID thrombosis.^27^ Furthermore, the rate of maternal death among infected mothers was 9.6% (10/104) compared to non-death in the control group. Several articles reported a high rate of maternal mortality in Iran due to COVID-19.^16^ The number of maternal deaths due to the pandemic is still rising in some countries, especially middle-income countries, such as Iran.^13^ However, as regression analysis was not performed, we cannot definitely make any conclusions about the effect of COVID-19 on these mentioned results.

 This study’s strength is providing a control group to compare the outcomes between the two groups. However, it has some limitations. First of all, the study’s retrospective design can cause potential bias. Secondly, although we considered rigorous approaches to choosing the control group, it is possible that some asymptomatic pregnant women were misclassified. Third, although this study’s sample size was relatively higher than many other studies, it is still small for some less common maternal and neonatal outcomes. For this reason, it was impossible to perform adjustments and regression for some outcomes. This makes it difficult to give a definite opinion about the effect of COVID-19 on various pregnancy outcomes in this study. Thus, conducting more studies using control groups with much higher sample size is recommended to compare less prevalent but more serious outcomes. Moreover, following mothers with COVID-19 that continue their pregnancies is suggested to achieve documented data on the impact of COVID-19 infection on long-term maternal and neonatal outcomes.

 In conclusion,this study demonstrated maternal and neonatal adverse outcomes compared with those in the non-infected group. COVID-19 infection is associated with higher rates of CS, PTL, neonatal admission to the NICU, neonatal respiratory distress, and longer duration of hospitalization. Vertical transmission is also possible. Given the effect of many potential factors on pregnancy outcomes, using an adequate sample size is reasonable to provide more precise and comprehensive results. The results of this study help to provide more information on COVID-19 in pregnancy.
